# Super-Resolution Microscopy Reveals Presynaptic Localization of the ALS/FTD Related Protein FUS in Hippocampal Neurons

**DOI:** 10.3389/fncel.2015.00496

**Published:** 2016-01-12

**Authors:** Michael Schoen, Jochen M. Reichel, Maria Demestre, Stefan Putz, Dhruva Deshpande, Christian Proepper, Stefan Liebau, Michael J. Schmeisser, Albert C. Ludolph, Jens Michaelis, Tobias M. Boeckers

**Affiliations:** ^1^Institute for Anatomy and Cell Biology, Ulm UniversityUlm, Germany; ^2^Institute of Biophysics, Ulm UniversityUlm, Germany; ^3^Department of Neurology, Ulm UniversityUlm, Germany; ^4^Institute of Neuroanatomy, Eberhard Karls University TübingenTübingen, Germany

**Keywords:** FUS, RNA-binding proteins, local translation, amyotrophic lateral sclerosis, frontotemporal dementia, super-resolution microscopy, STORM, synapse

## Abstract

Fused in Sarcoma (FUS) is a multifunctional RNA-/DNA-binding protein, which is involved in the pathogenesis of the neurodegenerative disorders amyotrophic lateral sclerosis (ALS) and frontotemporal dementia (FTD). A common hallmark of these disorders is the abnormal accumulation of mutated FUS protein in the cytoplasm. Under normal conditions FUS is confined to the nuclear compartment, in neurons, however, additional somatodendritic localization can be observed. In this study, we carefully analyzed the subcellular localization of endogenous FUS at synaptic sites of hippocampal neurons which are among the most affected cell types in FTD with FUS pathology. We could confirm a strong nuclear localization of FUS as well as its prominent and widespread neuronal expression throughout the adult and developing rat brain, particularly in the hippocampus, the cerebellum and the outer layers of the cortex. Intriguingly, FUS was also consistently observed at synaptic sites as detected by neuronal subcellular fractionation as well as by immunolabeling. To define a pre- and/or postsynaptic localization of FUS, we employed super-resolution fluorescence localization microscopy. FUS was found to be localized within the axon terminal in close proximity to the presynaptic vesicle protein Synaptophysin1 and adjacent to the active zone protein Bassoon, but well separated from the postsynaptic protein PSD-95. Having shown the presynaptic localization of FUS in the nervous system, a novel extranuclear role of FUS at neuronal contact sites has to be considered. Since there is growing evidence that local presynaptic translation might also be an important mechanism for plasticity, FUS – like the fragile X mental retardation protein FMRP – might act as one of the presynaptic RNA-binding proteins regulating this machinery. Our observation of presynaptic FUS should foster further investigations to determine its role in neurodegenerative diseases such as ALS and FTD.

## Introduction

During the last decade a growing number of RNA-binding proteins has been identified as key players in the pathogenesis of amyotrophic lateral sclerosis (ALS) and frontotemporal dementia (FTD). Both diseases are now considered to be tightly linked by common clinical as well as pathophysiological hallmarks. ALS is a fatal, progressive neurodegenerative disorder which is characterized by the selective demise of motor neurons in the brain stem and spinal cord. FTD is the second most common form of early onset dementia leading to aggravating changes in personality and language. It is characterized by progressive neuronal loss in frontal and temporal lobes. While many ALS patients suffer from motor deficits, the majority of FTD patients develop cognitive and behavioral abnormalities. A common hallmark of both neurodegenerative disorders is the presence of pathologic protein deposits in the cytoplasm of affected neurons. In 2006, the RNA-binding protein TDP-43 encoded by the *TARDBP* gene was identified as a major component of ubiquitinated aggregates in ALS and FTD (Arai et al., 2006; Neumann et al., 2006). Subsequently, several mutations in the *TARDBP* gene have been reported as a primary cause of ALS (Gitcho et al., 2008; Kabashi et al., 2008; Sreedharan et al., 2008; Van Deerlin et al., 2008; Yokoseki et al., 2008). The identification of TDP-43 as an important protein in ALS pathogenesis directly triggered the discovery of ALS and FTD causing mutations in another RNA-/DNA-binding protein called Fused in Sarcoma (FUS) (Kwiatkowski et al., 2009; Vance et al., 2009; Blair et al., 2010; Damme et al., 2010). Similar to TDP-43, FUS is a ribonuclear protein that is mainly located in the nucleus but was found to shuttle between the nucleus and the cytoplasm (Fujii and Takumi, 2005; Fujii et al., 2005). ALS-linked mutations in FUS are mainly found in the NLS (nuclear localization signal), giving rise to the abnormal presence of FUS in the cytoplasm. Interestingly, increasing levels of cytoplasmic FUS seem to be associated with a more aggressive disease course (Dormann et al., 2010; Waibel et al., 2013). In FUS associated FTD neuronal abnormalities are found in various areas within the central nervous system (CNS) including the hippocampus (Mackenzie et al., 2010). While pathologic FUS aggregates in ALS only occur in the presence of FUS mutations, no FUS mutations have been reported in FTD cases with FUS pathology.

Since a clear correlation between abnormal subcellular localization and disease progression is a unique feature of FUS mediated neurodegeneration, it is pivotal to determine the exact intracellular distribution of this protein in healthy neurons. FUS may not just be a nuclear protein but is also transported along neuronal processes to synapses in response to the specific and tightly controlled demands of neuronal plasticity and activity. For instance, FUS was also shown to be involved in nuclear messenger ribonucleic acid (mRNA) export and dendritic mRNA transport (Fujii and Takumi, 2005; Fujii et al., 2005) and recently its presence in granules along dendrites in the mouse and human CNS including the spinal cord (Aoki et al., 2012) was demonstrated. Cortical neurons from FUS mutant mice and cultured hippocampal neurons from FUS deficient mice display disturbed spine maturation and excessive dendritic branching (Fujii and Takumi, 2005; Fujii et al., 2005; Sephton et al., 2014).

Intriguingly, besides their postsynaptic and/or dendritic localization, mRNAs as well as RNA-binding proteins have also been detected in the axonal compartment with a supposed function in development- and activity-dependent plasticity of the outgrowing neurite. Among those are fragile X mental retardation protein (FMRP; Antar et al., 2006; Li et al., 2009), survival motor neuron protein (SMN; Rossoll et al., 2003; Liu et al., 2011), heterogeneous ribonucleoprotein R (hnRNP R; Glinka et al., 2010) and TDP-43 (Godena et al., 2011; Fallini et al., 2012) giving rise to the supposition that local mRNA translation is also realized in presynaptic axon terminals (Hörnberg and Holt, 2013). Axonal pathology is supposed to play a critical role in motor neuron diseases (Jablonka et al., 2004).

An important open question is the precise subcellular localization of FUS, which has thus far only been addressed by light microscopy after immunolabeling. However, in general, conventional fluorescence microscopic analysis of proteins localized to small subcellular compartments – like synapses – are limited by the Abbe’s law to a resolution of 200–300 nm even with confocal microscopes, restricting the accuracy of the localization. Recent approaches in the field of super-resolution microscopy can overcome this barrier and provide a powerful tool for life sciences to analyze functional structures such as multiprotein complexes with typical diameters smaller than 50 nm (Betzig, 2015; Hell, 2015). In this study we used single molecule localization microscopy (SMLM) (Rust et al., 2006; Fölling et al., 2008; Gunkel et al., 2009) by using stochastic on/off cycles of organic dye molecules, oftentimes also referred to as direct stochastic optical reconstruction microscopy (dSTORM) (Heilemann et al., 2008) to determine the exact intracellular localization of FUS protein compared to post- and presynaptic markers in hippocampal neurons. Previously, a related technique has been applied to the localization of common synaptic proteins (Dani et al., 2010) or to localize the neurotrophic receptor BDNF (brain derived neurotrophic factor) in the synapse (Andreska et al., 2014), thereby validating our approach. We found that FUS is located in axon terminals in close apposition to the presynaptic scaffolding protein Bassoon and co-localizing with the synaptic vesicle protein Synaptophysin1 (SYP1).

## Materials and Methods

### Animal Ethics Statement

All animal experiments were performed in compliance with the guidelines for the welfare of experimental animals issued by the Federal Government of Germany, the National Institutes of Health and the Max Planck Society. The experiments in this study were approved by the review board of the Land Baden-Württemberg, Permit Number Nr. O.103.

### *In Situ* Hybridization

*In situ* hybridization was performed as described previously (Proepper et al., 2011). Transcripts were detected with S^35^ labeled cDNA antisense oligonucleotides. FUS mRNA (bp 1067–1100) (GenBank:NM_001012137.1) was detected using the oligonucleotide 5′-GTC GAT TGC AGC TTT AGC AGA AGG TGG GTC ATC A-3′. Oligonucleotides were purchased from MWG-Biotech (Ebersberg, Germany).

### Immunohistochemistry

#### Immunolabeling of Rat Brain Slices for Conventional Fluorescence Microcopy

Adult Sprague-Dawley rats were perfused and fixed with 4% paraformaldehyde and 0.5% glutaraldehyde diluted in PBS. After fixation, brains were cut into 80 μm sagittal slices using a vibratome. The sections were then treated with 1% sodium borohydride in PBS for 5 min, followed by a washing step in PBS. For permeabilization, free-floating slices were incubated in 0.5% Triton X-100 in PBS for 15 min. After additional three washing steps the slices were blocked with 10% horse serum in PBS for 4 h at 4°C. After blocking, primary antibodies were applied overnight at 4°C in 5% horse serum in PBS. The slices were washed three times before the secondary antibodies were applied. After incubation for 1 h at room temperature, the sections were counterstained with 4′,6-diamidino-2-phenylindole (DAPI) in PBS and washed another three times. For fluorescence microscopy the slices were mounted using Vecta Mount AQ (Vector Laboratories, USA).

#### Immunoperoxidase Stainings

Whole rat brains at the indicated age were fixed in Bouins solution for 48 h and then embedded in paraffin. Sections were then de-paraffinized and the endogenous peroxidase activity was inhibited with a solution (10% methanol and 0.03% H_2_O_2_ in PBS). Afterwards, sections were washed three times with PBS and permeabilized for 15 min with 0.5% Triton X-100 in PBS. After 2 h of blocking with 2% bovine serum albumin (BSA) in PBS the sections were incubated with primary antibodies in PBS overnight at 4°C. To visualize the immunoreactivity the avidin-biotin-peroxidase technique was used. Therefore biotin-labeled secondary antibodies diluted in PBS with 0.5% BSA were added and incubated for 2 h at room temperature. After this step, the sections were incubated for another 2 h with a biotin peroxidase-streptavidin complex. The peroxidase reactivity was detected by adding 0.02% DAB (3,3′-Diaminobenzidine) in 50 mM Tris HCl pH 7.5. After dehydration the sections were mounted with Entellan^®^ (Merck, Germany).

### Cultured Cells

#### Cell Lines

NIH3T3 cells (Deutsche Sammlung von Mikroorganismen und Zellkulturen, DSMZ) were maintained in DMEM (Dulbecco’s modified Eagle’s medium, Invitrogen, Germany), supplemented with 10% FCS (fetal calf serum) and 100 units/ml penicillin/streptomycin (Invitrogen). All cells were cultured in a humidified atmosphere with 5% CO_2_ at 37°C.

#### Culturing Rat Hippocampal Neurons

Hippocampal cell culture from rat embryos was performed as previously described with minor modifications (Boeckers et al., 2005). In brief, time mated adult pregnant Sprague-Dawley rats were anesthetized and sacrificed by CO_2_ narcosis and subsequent decapitation after sufficient hypnosis. Embryos were removed from the uterus 18–19 days after mating. Brains were dissected out from the embryos and placed into ice-cold HBSS (Hank’s balanced salt solution). After isolation of the hippocampi, cells were isolated by trypsin digestion and mechanical dissociation. The hippocampal neurons were then plated on coverslips coated with poly-L-lysine 0.05–0.1 mg/ml (Sigma–Aldrich, Germany) on 12- or 24-well plates. Cells were grown in Neurobasal medium supplemented with B27, L-glutamine (2 mM, gibco), and 100 units/ml penicillin/streptomycin (Invitrogen). Cells were grown at 37°C in a humidified atmosphere containing 5% CO_2_. At 14 days after seeding cells were prepared for immunocytochemistry.

#### Immunolabeling of Rat Hippocampal Neurons for SMLM and Conventional Fluorescence Imaging

All described washing steps were performed with PBS (without calcium and magnesium). Prior to the fixation, cells were washed 4x with PBS. Subsequently, cells were incubated with pre-chilled methanol for 5 min at -20°C. The fixation was followed by three washing steps 5 min each.

Following steps were performed on a shaker. The samples were prepared for antibody incubation with a buffer for blocking and permeabilization (3% BSA, 0.1–0.3% Triton X-100 in PBS) for min 2 h. Primary antibodies were diluted in the blocking/permeabilization buffer and then cells were incubated for 2–3 days at 4°C (primary antibodies see **Table 1** at the end of section “Material and Methods”). Thereafter, coverslips were washed 3x 30 min at room temperature and then incubated with the secondary antibody in blocking/permeabilization buffer for 2–3 h at room temperature. Dye labeled secondary antibodies were purchased from Invitrogen (derived from goat, Alexa Fluor^®^ 532 and 647 for SMLM, Alexa Fluor^®^ 488, 568, and 647 for conventional fluorescence microscopy, all diluted 1:750). Cells were washed again 3x 30 min. Coverslips for super resolution microscopy were then immediately used for analysis or for longer storage cells were post-fixated with methanol as described above. For conventional imaging coverslips were briefly washed in deionized water after the last PBS washing step and mounted in Vecta Mount containing DAPI in a dilution of 1:50.000.

**Table 1:** Primary antibodies.

Antibody	RRID citation	Dilution and Application
Bassoon(C)^∗^ Bassoon(N)^∗^ FUS FUS FUS GAD65 GAPDH Gephyrin GFAP GluA1 Homer1b/c	Synaptic Systems GmbH Cat# 141 003 RRID:AB_887697 Enzo Life Sciences Cat# ADI-VAM-PS003 RRID:AB_10618753 Proteintech Group Cat# 11570-1-AP RRID:AB_2247082 Santa Cruz Biotechnology Cat# sc-47711 RRID:AB_2105208 Sigma–Aldrich Cat# HPA008784 RRID:AB_1849181 Abcam Cat# ab85866 RRID:AB_1860505 Abcam Cat# ab9484 RRID:AB_307274 Synaptic Systems GmbH Cat# 147 011 RRID:AB_887717 Sigma–Aldrich Cat# G3893 RRID:AB_477010 Synaptic Systems Ca# 182 011 RRID:AB_1630258 Synaptic Systems GmbH Cat# 160022 RRID not available	1:500 (STORM) 1:500 (FI & STORM) 1:500 (FI on tissue sections) 1:400/1:200 (WB/DAB) 1:2000/1:500 (WB on NIH lysate/FI & STORM) 1:500 (FI) 1:1000 (WB) 1:500 (FI) 1:500 (FI) 1:500 (FI) 1:500 (STORM)
MAP2 MAP2	Sigma–Aldrich Cat# M4403 RRID not available Synaptic Systems Cat# 188 004 RRID:AB_2138181	1:500 (FI on tissue sections) 1:500 (FI on cultured neurons)
PSD-95 PSD-95 SYP SYP1 S-100 (ß) VGLUT1	Abcam Cat# ab2723 RRID:AB_303248 Synaptic Systems GmbH Cat# 124 011 RRID:AB_10804286 Abcam Cat# ab14692 RRID:AB_301417 Synaptic Systems GmbH Cat# 101 004 RRID:AB_1210382 Sigma–Aldrich Cat# S2532 RRID:AB_477499 Synaptic Systems Cat# 135 304 RRID:AB_887878	1:500 (FI & STORM) 1:1000 (WB) 1:3000 (WB) 1:500 (STORM) 1:500 (FI) 1:500 (FI)

*FI, conventional fluorescence imaging; DAB, 3,3′-Diaminobenzidine; WB, western blot. ^∗^(C) and (N), C-terminal or N-terminal antigenic epitopes, respectively.*

### Image Acquisition, Processing, and Analysis

#### Conventional Fluorescence Microscopy – Image Acquisition

Images were obtained with an upright fluorescence microscope (Zeiss Axioskop 2 and Zeiss Imager.Z1 with an apotome, Zeiss, Germany). Pictures were taken with the Axiovison 4.7.1 software (Zeiss, Germany) and analyzed with ImageJ 1.46r (National Institutes of Health of the United States; RRID:nif-0000-30467).

#### Super-Resolved Single Molecule Localization Microscopy – Image Acquisition

In order to obtain super-resolution 2D images of double labeled synapses the SMLM technique dSTORM was used (primary antibodies see **Table 1**). Experiments were performed on a home built microscope. For illumination an objective-type TIRF (total internal reflection fluorescence) configuration with an oil-immersion objective (APO TIRF 60x, NA 1.49 Oil, Nikon) was used. Three continuous-wave laser sources (640 and 402 nm, Toptica Photonics, Germany, and 532 nm, Cobolt, Sweden) were combined, controlled in intensity by an AOTF (acousto-optical tunable filter; Gooch & Housego, United Kingdom) and used for excitation and activation. Single molecule fluorescence signals were separated from activation and excitation light as well as split into two detection channels by using appropriate dichroics and emission filters (AHF analysentechnik, Tübingen, Germany) and finally passed on to two halves of an EMCCD camera (iXon 897, Andor Technology, United Kingdom) with an effective pixel size of 132 nm. For dual color images a series of 30.000 images was acquired for the red channel, followed by another 30.000 images for the green channel, each taken at a frequency of 46 Hz with typical laser intensities of 0.4–0.7 kW/cm^2^ for excitation and 0–3 W/cm^2^ for activation. Microscope drift in the axial, i.e., *z*-direction was automatically corrected during image acquisition by a home built autofocus system. The xy-drift was highly reduced by instrument design and was therefore oftentimes negligible or could be compensated as described later.

For dSTORM imaging, cells were placed in a degassed PBS imaging buffer containing 100 U/mL glucose oxidase, 400 U/mL catalase, 4% (wt/vol) glucose and 100 mM cysteamine (all from Sigma–Aldrich, Schnelldorf, Germany) at pH 7.5.

#### Single Molecule Localization Microscopy – Data Processing

Acquired images were analyzed using custom written Matlab software (MathWorks, Natick, MA, USA). In short, the image series was background corrected by applying a running temporal median filtering (Hoogendoorn et al., 2014) with a window size of 101 frames. The center position of fluorescent spots above a certain threshold was identified by their COM and filtered according to intensity, width and asymmetry criteria. Fluorophore localizations were reconstructed in a pixel raster of 10 nm and weighted with the intensity of the individual localization. The second channel of a two color image was transformed onto the first channel according to a transformation function obtained by imaging fluorescent beads (TetraSpeck Microspheres, life technologies, Darmstadt, Germany) in both color channels. If xy-drift was not negligible it was compensated by applying a redundant cross correlation algorithm to the identified localizations (Wang et al., 2014). All presented super-resolution reconstructed images were processed with a Gaussian blur (sigma = 10 nm) to account for the localization precision.

#### Single Molecule Localization microscopy – Image Analysis

Further analysis of reconstructed super-resolution images was done with ImageJ under blinded conditions for statistical evaluation. Synapses were identified manually in two color images as co-localizing or near-by-localizing bar-like structures and marked by a 300 nm wide line, perpendicular to the apparent synaptic cleft. Intensities where averaged over the line width yielding a one-dimensional intensity distribution along the line. The COM is calculated by taking the arithmetic mean of the labeled protein positions along the marked transsynaptic axis weighted by their respective intensities. The distance of two individual protein populations is then given by the distance of the COMs of the two underlying intensity profiles (performed always on raw data without applying a Gaussian blur). The value of many such single synapse measurements is then entered into a histogram for the respective protein pairs. The reported mean distance of two proteins results from taking into account only the 40% of all synapses with the largest distances corresponding to the largest entries in the respective histograms. This step was introduced to reduce the ambiguity that arises from imaging a three dimensional structure in only two dimensions. As therefore the orientation of the synapses with respect to the imaging plane is unknown, two clearly separate protein distributions (in 3D) could appear in 2D as closely co-localizing distributions with a false too small COM distance if the plane of the synaptic cleft is parallel to the imaging plane. The more the synaptic cleft is tilted out of the imaging plane the larger the measured distance will be. By restricting the data to the largest measured distances one ensures that mainly synapses whose synaptic cleft is oriented (close to) perpendicular to the imaging plane are counted and therefore more accurate measurements are obtained. However, the reported results are not affected by the particular cut-off chosen (see Supplementary Figure S3 for different cut-offs).

For all stainings including FUS also the COM in two dimensions was determined, in contrast to the COM in one dimension along a line as described before. For this purpose synapses were identified in two color images manually as co-localizing protein distributions and marked by a rectangle enclosing these distributions. The COMs in two dimensions for each protein population inside the rectangle and their respective Euclidean distance were calculated. Results were again grouped in a histogram as described above.

### Protein Biochemistry

#### Tissue Fractionation

Subfractionation of rat brain tissue was performed as described previously with some modifications (Distler et al., 2014). All steps were performed at 4°C or on ice. In brief, tissue was homogenized in homogenization buffer (320 mM sucrose, 5 mM HEPES, pH 7.4, protease inhibitor mixture (Roche, Germany). To remove cell debris and nuclei the homogenate was centrifuged for 10 min at 1.000 × *g*. The supernatant (S1) was centrifuged for 20 min at 12.000 × *g* to obtain the soluble fraction (S2) and the crude synaptosomal fraction (P2). For further fractionation, P2 was resuspended in Tris buffer (320 mM sucrose, 5 mM Tris/HCl pH 8.1) and centrifuged in a sucrose density gradient (0.8 M/1.0M/1.2 M sucrose) for 2 h at 200.000 × *g* and 4°C. The myelin-enriched fraction (My) was obtained from the top of the gradient, the light membranes (LM1) from the 0.8/1.0 interphase, and the purified synaptosomal fraction (Sy) from the 1.0 M/1.2 M interphase. The synaptosomes were then diluted in five volumes of 1 mM Tris pH 8.1 and stirred on ice for 30 min followed by another centrifugation step for 30 min at 33.000 × *g*. The resulting pellet P3 was re-suspended in 5 mM Tris pH 8.1 and further fractionated by centrifugation on another sucrose gradient (0.8 M/1.0 M/1.2 M sucrose) for 2 h at 200.000 × *g*. The 1.0 M/1.2 M interphase (containing the synaptic junctions, SJ) was collected and resuspended in Triton buffer (320 mM sucrose, 5 mM Tris pH 8.1 and 0.5% Triton X-100). After stirring on ice for 15 min another centrifugation step for 30 min at 33.000 × *g* was performed resulting in a last pellet containing the one-Triton extracted postsynaptic density (PSD) fraction.

#### Western Blot

Protein samples were diluted in SDS (sodium dodecyl sulfate) loading buffer. Protein concentration was determined by amido black assays. Samples were separated by SDS-PAGE (polyacrylamide gel electrophoresis) and subsequently blotted on PVDF membranes (GE Healthcare). Immunoreactivity of primary antibodies (see **Table 1**) was detected using HRP-conjugated secondary antibodies and the Pierce^®^ ECL detection kit (Thermo Scientific).

## Results

### FUS in Non-neuronal Cells

**FIGURE 1:**
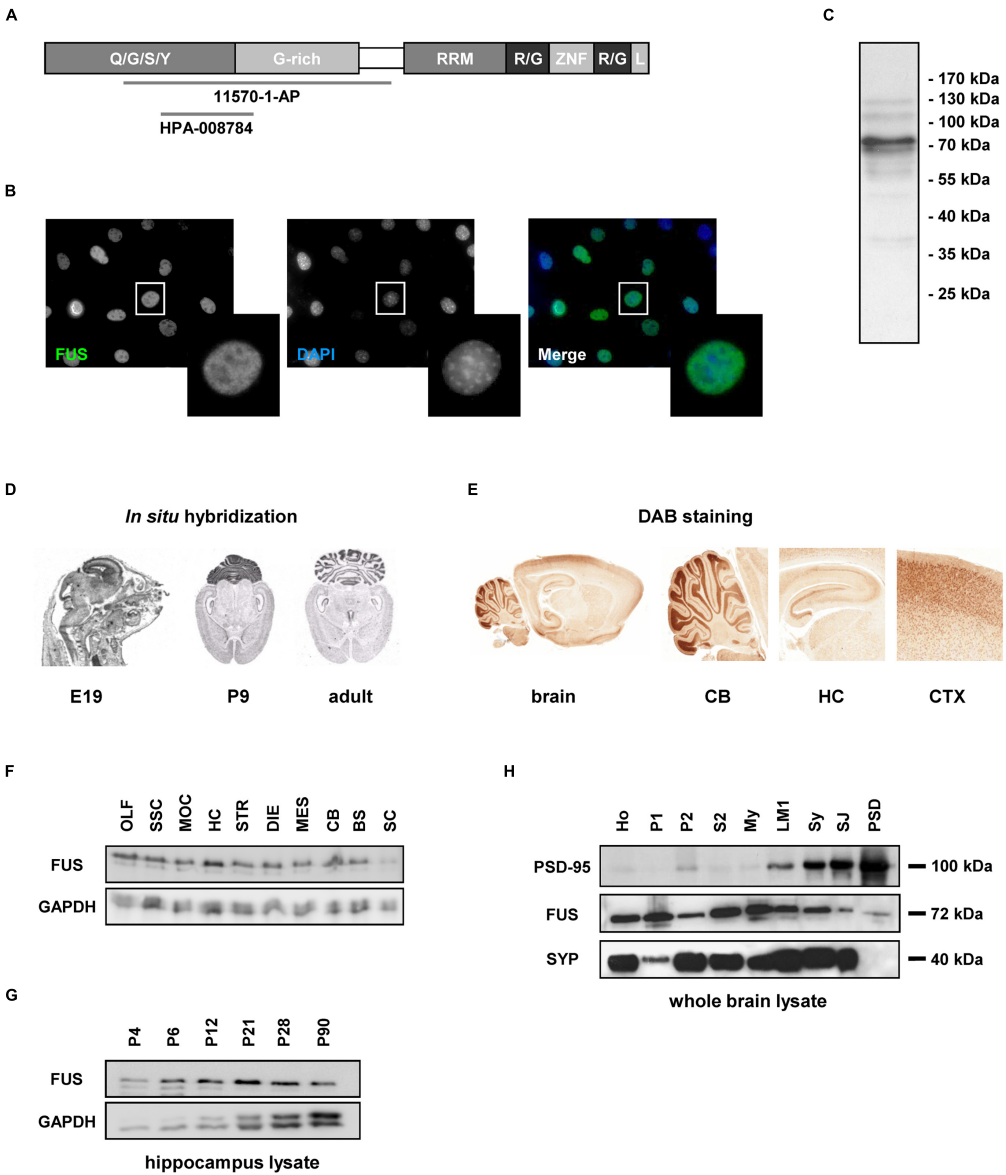
**Fused in Sarcoma (FUS) expression in non-neuronal cells and in the rat CNS. (A)** Domain architecture of FUS and available epitope regions of antibodies. Human FUS consists of 526 amino acids and comprises an N-terminal QGSY rich region, a Glycine rich region, a RNA recognition motif, two R/G rich domains, a zinc finger motif and a nuclear localization signal. Q/G/S/Y = glutamine/glycine/serine/tyrosine, G-rich = glycine rich region, RRM = RNA recognition motif, R/G = arginine/glycine, ZNF = zinc finger, L = nuclear localization signal. **(B)** Immunostaining of FUS in NIH3T3 cells. FUS is predominantly localized in the nuclear compartment counterstained with DAPI. **(C)** Western blot of NIH lysate. FUS antibody detects a 72 kDa band. **(D)** *In situ* hybridization of FUS during brain development. The mRNA is mainly expressed in the cortex, the hippocampus and the cerebellum. No obvious dendritic localization of FUS mRNA can be observed. E19 = embryonic day 19, P9 = postnatal day 9. **(E)** DAB immunostainings of FUS of a P12 rat brain. The observed expression pattern with high abundance of the protein in the hippocampus, cortex and the granular cell layer of the cerebellum is in good accordance to the expression pattern observed by *in situ* hybridization experiments on P9. CB, cerebellum; HC, hippocampus; CTX, cortex. **(F)** Western blot analysis of FUS expression in the rat CNS at P12. FUS is abundant in all of the CNS regions investigated but with only a weak signal in the spinal cord. The relatively highest concentration can be observed in the hippocampus. OLF, olfactory bulb; SSC, somatosensory cortex; MOC, motor cortex; HC, hippocampus; STR, striatum; DIE, diencephalon; MES, mesencephalon; CB, cerebellum; BS, brain stem; SC, spinal cord. GAPDH was used as a loading control. **(G)** Developmental stages were examined in homogenates of the hippocampus at different time points. In this specific brain region the expression of the protein is quite stable during development with a peak at postnatal day P21. **(H)** Biochemical analysis of subcellular fractions from adult rat whole brain. FUS was detected in all subfractions including purified synaptosomes, synaptic junctions and the PSD fraction. PSD-95 and SYP antibodies were used to control fractionation procedure. PSD-95 shows a clear enrichment toward the PSD fraction, while SYP was not detected in the PSD fraction indicating a high grade of PSD purification. Ho, homogenate; P1, nuclear-enriched fraction; P2, crude synaptosomes; S2, soluble fraction; My, myelin; LM1, light membranes; SY, purified synaptosomes; SJ, synaptic junctions; PSD, postsynaptic density fraction.

First, non-neuronal NIH3T3 cells were stained with an antibody against the FUS N-terminus (diagram showing the epitope is represented in **Figure 1A**, see also **Table 1**) using DAPI as nuclear counterstain. FUS was expressed endogenously and predominantly in the nucleus overlapping with DAPI (**Figure 1B**). This FUS antibody has already been thoroughly characterized (Munoz et al., 2009; Neumann et al., 2009; Urwin et al., 2010; Aoki et al., 2012). In Western blot with NIH3T3 cell lysates (mouse embryonic fibroblast cell line) FUS was clearly detectable as a 72 kilodalton (kDa) protein (**Figure 1C**).

### Expression of FUS in the Rat CNS

Next, we analyzed FUS expression in the rat CNS using *in situ* hybridization. We detected FUS mRNA in all brain areas, especially in the cortex, the hippocampus, the granular cell layer of the cerebellum and in the spinal cord. The sharply bound lines in the hippocampus indicate a predominantly somatic localization. These expression patterns could be observed at all developmental stages investigated (**Figure 1D**).

Immunohistochemical DAB stainings of FUS in rat brain sections confirmed its broad expression throughout the rat brain (Supplementary Figure S1). The expression analysis of FUS in different brain regions was performed in postnatal day P12 tissue, since the protein was strongly expressed at this age (see **Figures 1D,G**). In accordance with the *in situ* hybridization data, FUS was highly abundant in the cortex, the hippocampus and the granular cell layer of the cerebellum (**Figure 1E**). For expression patterns in various brain regions at different neuronal developmental steps see Supplementary Figure S1. Western blot analysis of tissue lysates (homogenates at postnatal day P14) from different brain regions showed the abundant presence of the protein throughout the CNS (**Figure 1F**). This analysis also demonstrated remarkably high levels of FUS in the hippocampus. Interestingly, we found comparably low levels of FUS in the spinal cord. Next, we monitored closer the expression levels of FUS in the hippocampus at distinct stages of postnatal development (**Figure 1G**). The expression of FUS was sustained in the hippocampus and during all investigated developmental stages with highest levels at early developmental stages between P12 and P21 in relationship to the loading control protein GAPDH (glyceraldehyde 3-phosphate dehydrogenase). When analyzing subcellular fractions from whole brain, the protein was found in the synaptosomal fraction (synaptic junctions) and in decreasing amount toward the PSD fraction in which only a faint band appeared (**Figure 1H**).

### Subcellular Localization of FUS in Cultured Hippocampal Cells

**FIGURE 2:**
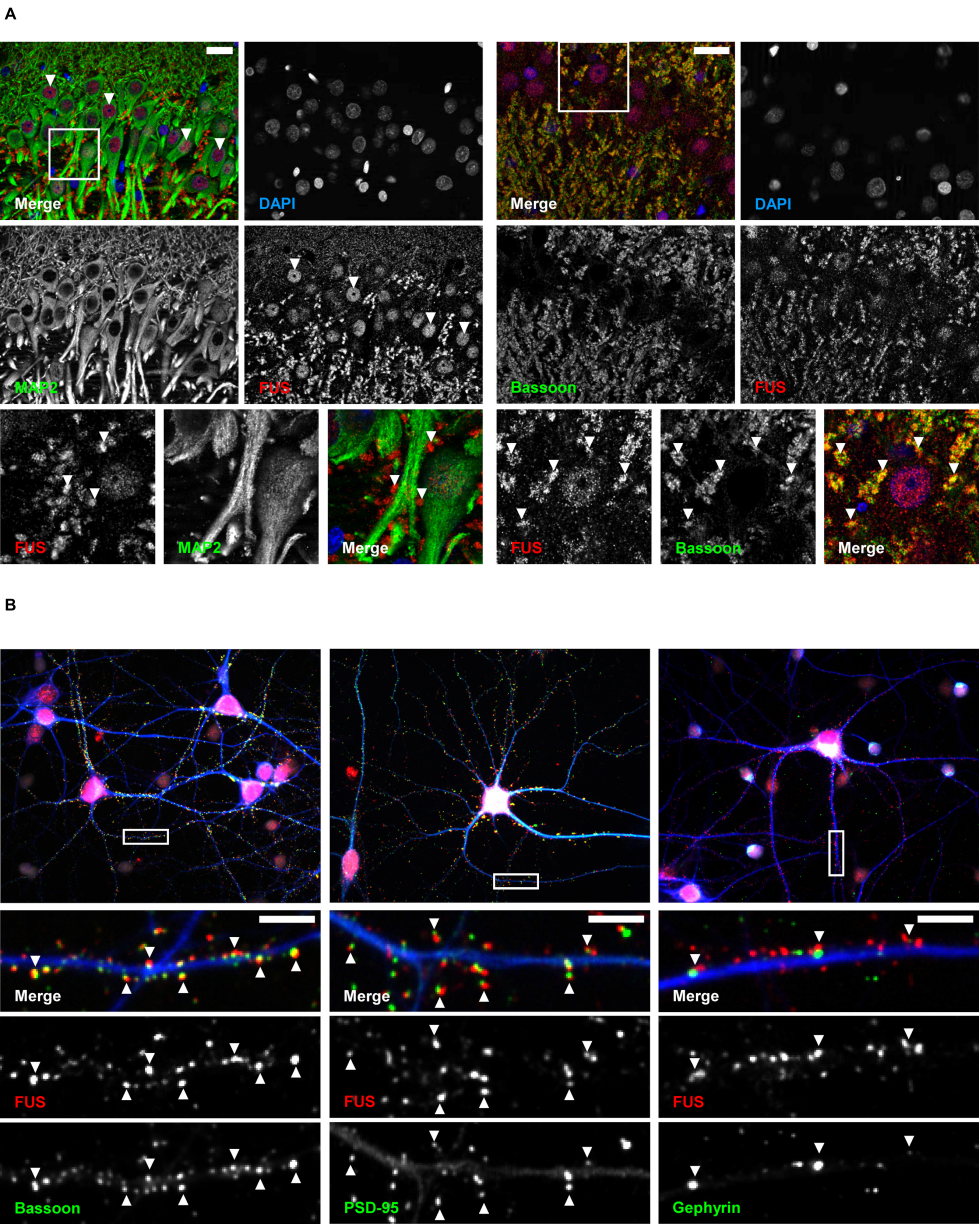
**Subcellular localization of FUS in hippocampal tissue and primary neurons. (A)** Immunofluorescence staining of the hippocampal CA1 region in an adult rat brain. The lower row depicts magnifications of rectangles indicated in the overview images above. FUS was found in the nuclei of hippocampal neurons (arrowheads in middle row, left) and was additionally localized in punctae alongside MAP2 positive dendrites (arrowheads in lower row, left). FUS positive punctae co-localized with the synaptic marker Bassoon (arrowheads in lower row, right). Scale bars represent 25 μm. **(B)** Localization of endogenous FUS in 14 days *in vitro* primary hippocampal neurons. Lower rows show magnifications as merged or single channels of rectangles in the overview images of the respective neurons in the first row. FUS could be clearly seen in the nucleus (overview images in red, nuclei stained with DAPI) and in a punctuated pattern alongside MAP2 (blue) positive dendrites. These punctae co-localized with the synaptic markers PSD-95 and Bassoon (arrowheads) but less with the inhibitory postsynaptic marker Gephyrin. Scale bars (white) represent 5 μm.

Next, we studied the subcellular localization of FUS in the hippocampus CA1 (cornu ammonis) region (**Figure 2A**). To that end, we performed immunohistochemical stainings on adult rat brain sections. Besides its localization in the nuclei, FUS was located in punctae alongside the MAP2 positive dendrites. FUS punctae were additionally co-localizing with the presynaptic scaffolding molecule Bassoon.

In line with our findings in brain sections of adult rats, a clear localization of FUS in the nuclei as well as along MAP2 positive dendrites was observed in cultured primary hippocampal neurons (**Figure 2B**). A high proportion of FUS clearly co-localized with the presynaptic marker Bassoon and the postsynaptic density protein 95 (PSD-95), an excitatory postsynaptic marker of glutamatergic synapses. Both markers have been used for the following super-resolution studies. Additionally, FUS co-localized to a lesser extent with the inhibitory postsynaptic marker Gephyrin. Supplementary Figure S2A demonstrates statistical evaluation of co-localizing FUS – PSD-95 vs. FUS – Gephyrin punctae. Only 14% of all FUS punctae in dendritic areas co-localize with Gephyrin whereas 65% can be found at PSD-95 accumulations. To further substantiate our observation, we stained FUS with glutamatergic pre- and postsynaptic markers VGLUT1 (vesicular glutamate transporter 1) and GluA1 (glutamate AMPA receptor subunit 1), respectively, and the GABAergic presynaptic marker GAD65 (65 kDa subunit of glutamate decarboxylase) (Supplementary Figure S2B). The results are in line with the previous observations that FUS is predominantly associated with excitatory synapses.

Nuclear FUS was not only found in neurons but also in glial cells marked with GFAP (glial fibrillary acidic protein) and S-100 (ß-subunit), in which as opposite to neurons FUS remained nuclear (Mori et al., 2012) (Supplementary Figure S2C).

### Super-Resolved Single Molecule Localization Microscopy Reveals Localization of FUS in Axonal Terminals

**FIGURE 3:**
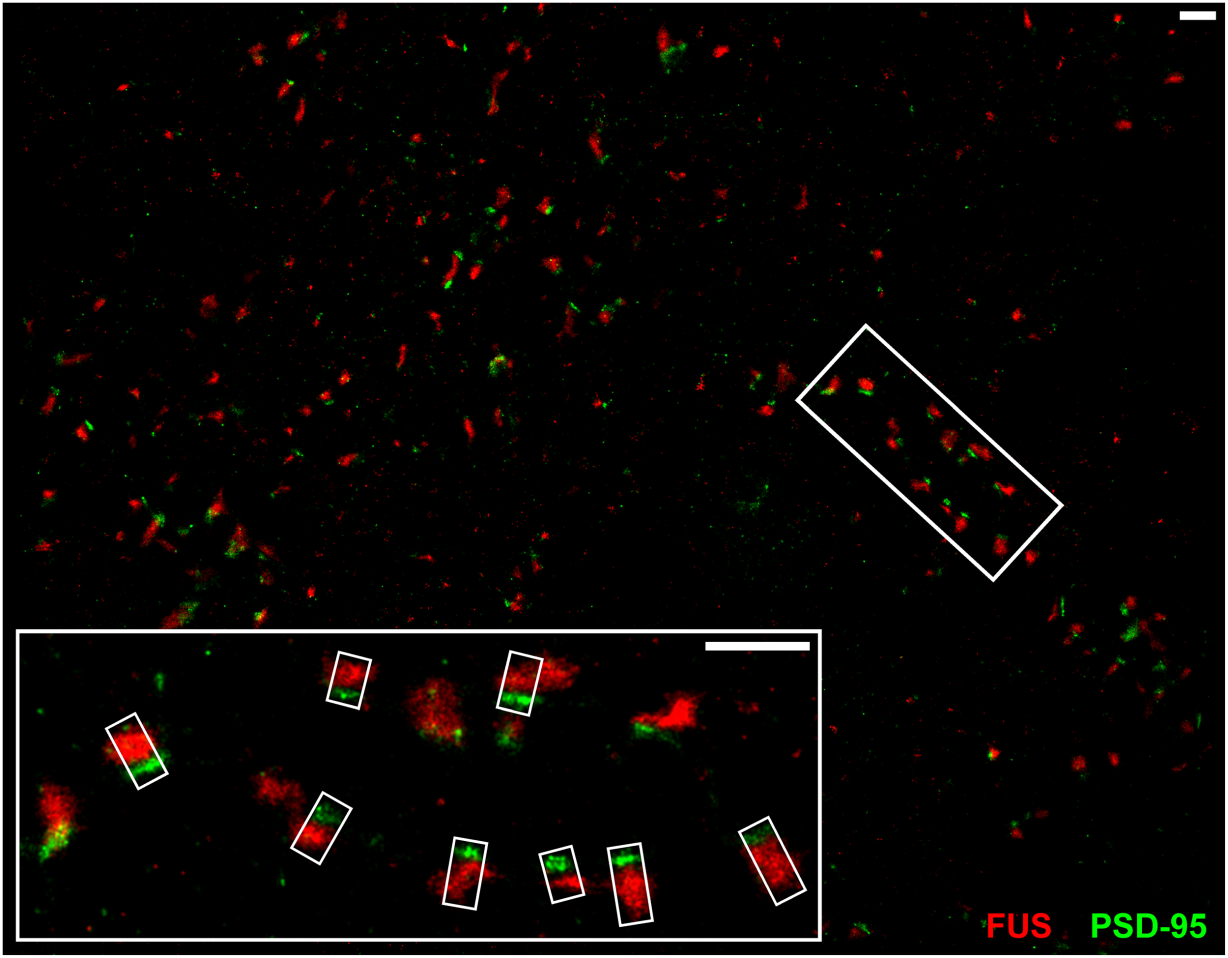
**SMLM imaging of near-by co-localizing FUS and PSD-95 in hippocampal neurons.** The upper image shows synapses of cultured hippocampal cells with immunolabeled FUS (red) and PSD-95 (green). The white rectangles in the magnification (lower image) represent exemplary lines for measuring with a defined width of 300 nm (short side of the rectangles) to receive an intensity profile along the transsynaptic axis. In this respect, the line spans the shortest distance between the two signals which is perpendicular to the bar-like distribution of PSD-95 and the apparent synaptic cleft. This is to calculate the distance of the COMs of each marker population. The scale bars (white) represent 1 μm.

Conventional fluorescence microscopy failed to resolve the exact localization of FUS within hippocampal synapses. In order to investigate the precise synaptic localization of FUS, a SMLM setup (described in Super-Resolved Single Molecule Localization Microscopy – Image Acquisition) with two excitation lasers (532 and 647 nm) was used. Immunolabeled FUS and reference synaptic proteins, namely SYP1, Bassoon and PSD-95, in cultured hippocampal neurons of two different preparations after 14 days *in vitro* were analyzed in this approach. At this stage of *in vitro* neuronal development it has been described that hippocampal neurons contain morphologically mature synapses (Grabrucker et al., 2009). In **Figure 3** a reconstructed super-resolution image of a co-staining of FUS and the postsynaptic marker PSD-95 is depicted. The magnification shows a presumable dendritic area with FUS opposed to PSD-95 signals.

**FIGURE 4:**
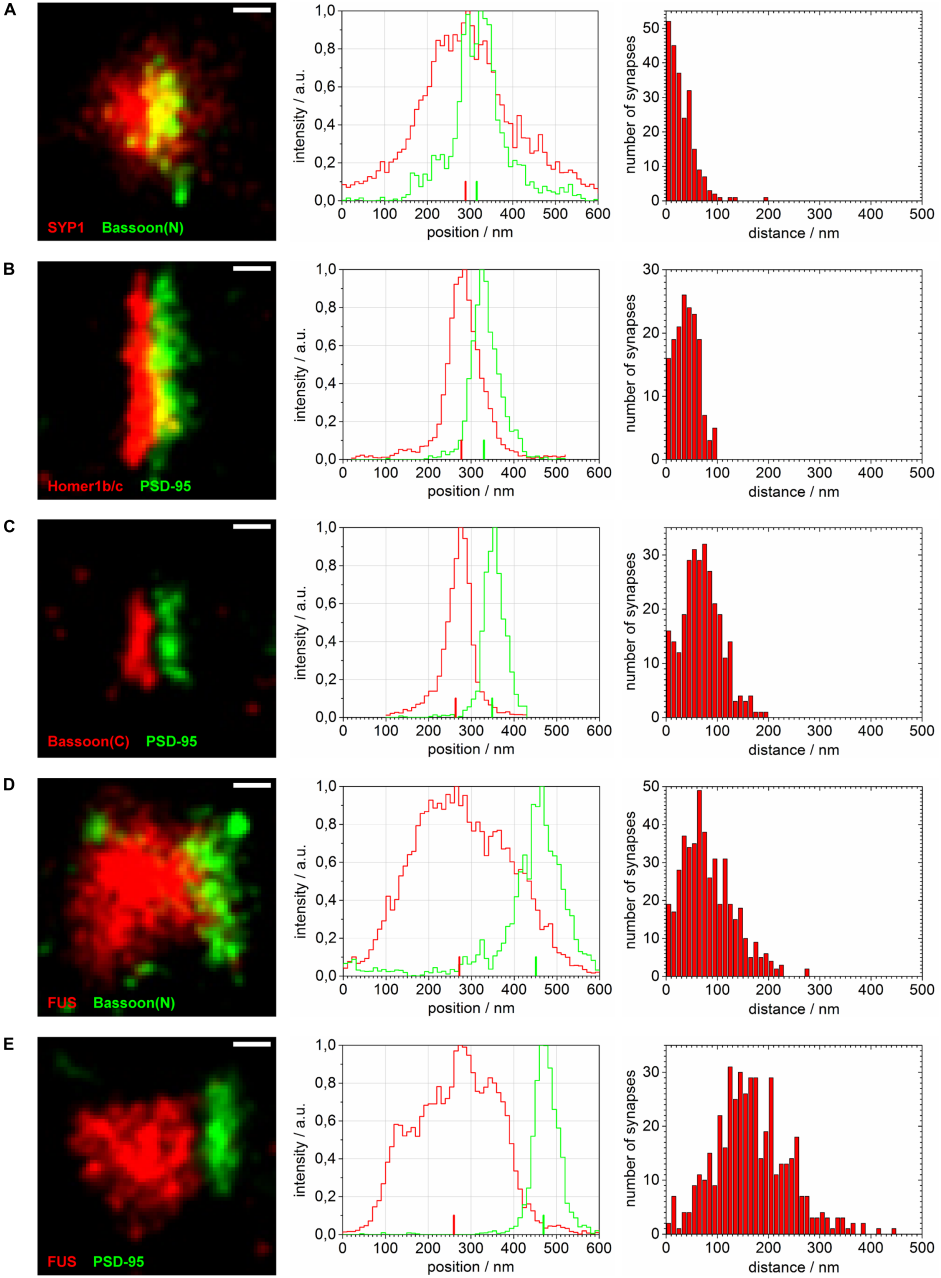
**Distance calculations of super-resolved synaptic protein populations.** Reconstructed super-resolved single molecule localization microscopy (SMLM) images of typical synapses (left), the corresponding raw intensity profiles without Gaussian blur (10 nm steps) along the transsynaptic axis (middle; respective COMs depicted as upright bars) as well as histograms of computed distances with 10 nm steps (right) are shown for: **(A)** SYP1 and Bassoon(N), **(B)** Homer1b/c and PSD-95, **(C)** Bassoon(C), and PSD-95, **(D)** FUS and Bassoon(N) and **(E)** FUS and PSD-95. The images are rotated with the transsynaptic axis in a horizontal orientation. White bars represent 100 nm (pixel size 10 nm).

Thereafter, to further analyze the precise localization of FUS within hippocampal synapses we used several synaptic markers as a reference. The aim was to identify the mean position of the distribution of FUS proteins on either the presynaptic side, the postsynaptic side or on both sides of the synapse along the transsynaptic axis (**Figure 4**). To achieve this, the intensity profiles of the protein distributions along the presumed transsynaptic axis were investigated (described in Single Molecule Localization microscopy – Image Analysis). The mean distance of FUS relative to the presynaptic marker Bassoon(N) (**Figure 4D**), as well as the mean distance of FUS relative to the postsynaptic marker PSD-95 were determined (**Figure 4E**). In addition, as control measurements, we determined the mean distance of typical pre- and postsynaptic proteins, as described below. The position of some of these markers relative to the synaptic cleft had been determined previously with super-resolution microscopy (Dani et al., 2010). We determined the distance between the two presynpatic markers Bassoon(C) and SYP1 (**Figure 4A**), between the two postsynaptic markers PSD-95 and Homer1b/c (**Figure 4B**), as well as between the presynaptic marker Bassoon(C) and the postsynaptic marker PSD-95 (**Figure 4C**). The determined distances are in good agreement with the previously published measurements.

The magnification in **Figure 3** shows exemplary selections (white boxes) used to obtain the intensity profiles along the presumed transsynaptic axis. From histograms of the computed COMs, the distance of the respective protein pairs was calculated (see Single Molecule Localization microscopy – Image Analysis). **Figure 4** displays typical synapses of each marker combination (left panel) and the corresponding raw intensity profiles along the transsynaptic axis including the respective COMs depicted as upright bars (middle panel). More than 150 synapses were measured for each combination. The distances of all synapses are grouped in a histogram in 10 nm steps to display the distribution of all measurements (right panel). The following mean distance values arise from imaging a three dimensional structure in two dimensions as described before and result from only taking into account 40% of all measured synapses with the highest distance to select for synapses whose synaptic cleft is tilted out of the imaging plane (for detailed explanation see Single Molecule Localization microscopy – Image Analysis). The combination of the two presynaptic proteins SYP1 and Bassoon(N) resulted in a mean distance of 55 ± 3 nm (standard error of the mean; *n* = 92 synapses) while Homer1b/c and PSD-95 showed a distance of 63 ± 2 nm (*n* = 66 synapses). A pre- and a postsynaptic marker combination with Bassoon(C) and PSD-95, respectively, lead to a calculated mean distance of 107 ± 2 nm (*n* = 117 synapses). Finally, FUS was analyzed versus Bassoon(N) and PSD-95 with mean distances of 133 ± 3 nm (*n* = 185 synapses) and 237 ± 4 nm (*n* = 181 synapses), respectively. The latter value highlights closer proximity of synaptic FUS to the presynaptic marker Bassoon in contrast to the postsynaptic marker PSD-95.

**FIGURE 5:**
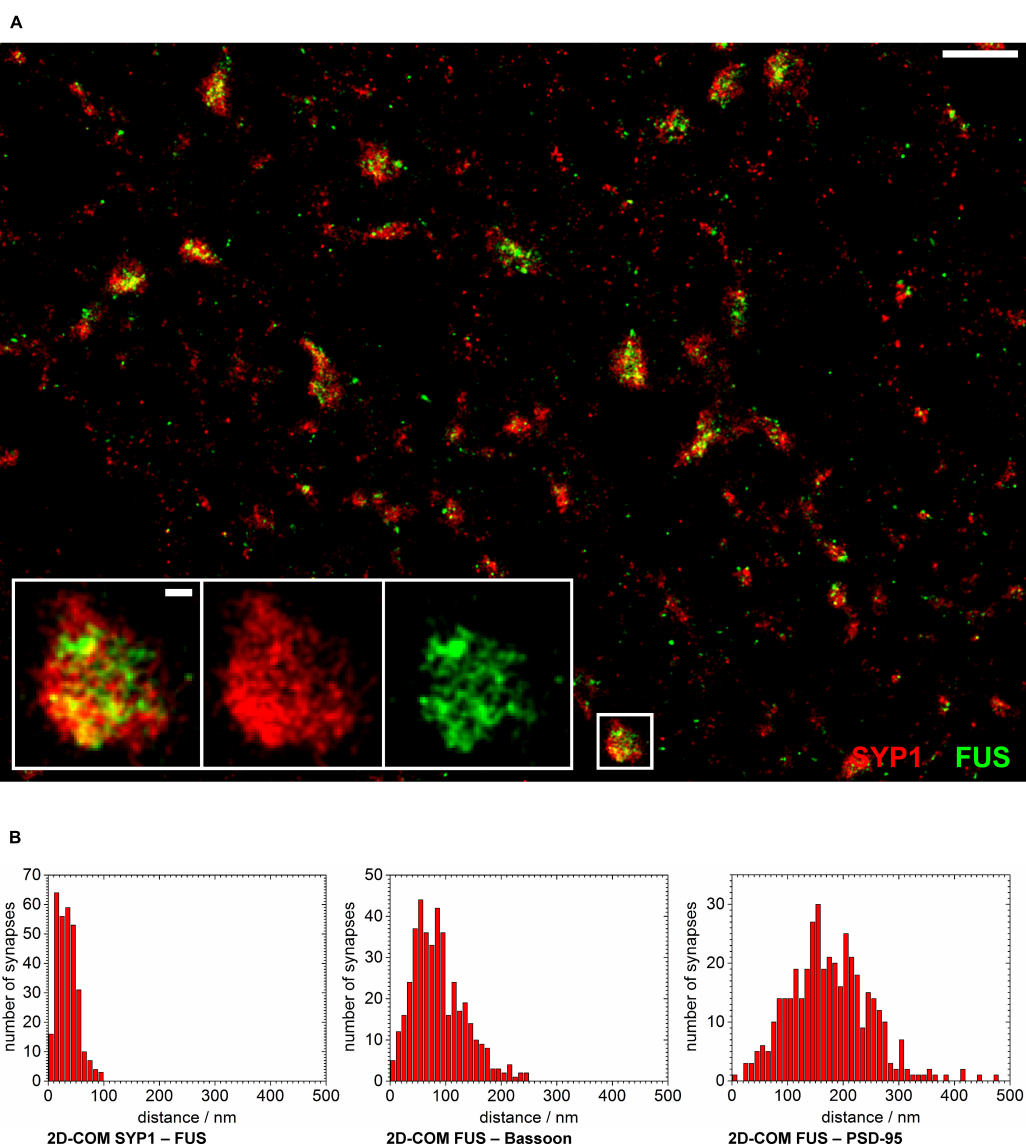
**FUS is closely localized to synaptic vesicles. (A)** The overview image shows synapses of cultured hippocampal neurons labeled with FUS (green) and SYP1 (red). The synaptic area marked with a white rectangle is magnified. Most synapses seem to show a distribution with SYP1 surrounding FUS signals in the ‘center.’ White scale bars represent 1 μm in the overview and 100 nm in the magnification. **(B)** The counted numbers of measured distances of the 2D-COM analysis are displayed as histograms with 10 nm steps.

In another approach we co-stained FUS with SYP1 in 14 days *in vitro* old neurons in order to investigate a localization of FUS at synaptic vesicles (see **Figure 5A**). We observed the highest co-localization of FUS with any of the tested synaptic markers with an almost complete overlap of the overall areas of labeled FUS and SYP1 proteins. Moreover, we find that firstly, both proteins seem to be less organized in bar-like structures in a ‘side’ view on a synaptic contact. They are rather disseminated in broader 2- and presumably 3-dimensional areas which can be observed as broader signals when measuring along the transsynaptic axis as described above (**Figures 4A,D,E**). Secondly, no apparent cleft between FUS and SYP1 could be observed, as between typical pre- and postsynaptic markers (**Figure 4C**). In conclusion, neither the shape of the protein distribution nor their relative positions allowed any inference to the orientation of the transsynaptic axis. Therefore, we determined the COMs in two dimensions in a rectangle that was drawn around a presumable synaptic structure with co-localizing FUS and SYP1 (described in Single Molecule Localization microscopy – Image Analysis). We obtained a value of 52 ± 1 nm (standard error of the mean; *n* = 122 synapses). However, it should be noted that this value does not correspond to an actual distance between the two proteins, but marks an upper estimate which is mainly given by the experimental uncertainty due to varying protein distributions of individual synapses and due to localization imprecisions. The experimental uncertainties cancel out if a clear separation between the positions of two protein distribution is seen, however, experimental uncertainties add up if the distributions of the two molecules coincide. To better classify this result we also applied this method on the other co-stainings including FUS (see above). FUS with the presynaptic marker Bassoon yielded 133 ± 3 nm (*n* = 168 synapses) while FUS with the PSD protein PSD-95 resulted in a mean distance of 246 ± 4 nm (*n* = 164 synapses; see **Figure 5B**). As expected, these results were minimally larger than those obtained with the one dimensional measurement along the transsynaptic axis.

## Discussion

### Main Findings

Our study on the differential expression of FUS in the rodent CNS revealed three main findings: (1) We could confirm and extend the current knowledge of a mainly nuclear localization of FUS and its prominent and widespread expression throughout the adult and developing rat brain, particularly in the hippocampus, the cerebellum and the outer layers of the cortex. (2) In hippocampal neurons, FUS was also present in synaptic compartments. (3) The SMLM analysis detected a presynaptic localization for FUS filling the axonal bouton in a very close proximity to synaptic vesicles.

### FUS Localization in the Rodent Central Nervous System

In line with previous reports, we found FUS to be strongly localized in nuclei throughout the rat brain (Huang et al., 2010). In particular, hippocampal, cerebellar and outer layer cortical neurons were rich in the expression of the protein. In FUS-associated FTD abnormalities of neurons are found in the cortex, the hippocampus, in lower motor neurons and to a lesser extent in the striatum, brainstem, and thalamus (Mackenzie et al., 2010). We chose the hippocampus as a model to study the exact localization of FUS as (1) previous work on FUS had mainly been done in hippocampal neurons (Belly et al., 2005; Fujii et al., 2005; Wang et al., 2008), (2) we found the highest expression of FUS in the hippocampus, and (3) for primary cell culture we chose a point in time for analysis that represents early developmental stages as we could observe FUS being highly expressed in immature rat brains. Furthermore, the hippocampus belongs to those brain regions affected in FTD with FUS and TDP-43 pathology (Mackenzie et al., 2010). When studying the CA1 region in hippocampal sections we found that FUS, together with a strong nuclear signal, showed a punctuated distribution along dendrites. FUS punctae co-localized with the synaptic marker Bassoon, suggesting a synaptic localization for FUS. In addition, the similar observations could be confirmed in primary hippocampal cells with FUS being in close proximity to the synaptic markers Bassoon and PSD-95. More detailed statistical evaluation revealed a strong co-localization of FUS with PSD-95, a protein which can be found at the postsynaptic side of glutamatergic synapses. Only a small amount of synaptic FUS co-localized with the inhibitory marker Gephyrin hinting toward a role also at a subpopulation of inhibitory synapses. Further studies will be needed to further investigate this association. As opposed to neurons, in glial cells FUS was only nuclear and this is in accordance with previous studies on human brain tissue (Mori et al., 2012). Moreover, FUS seems to rather cluster not directly at or beside the dendrite but at presumable dendritic spine specializations in the vicinity of postsynaptic densities of excitatory synapses (Boeckers, 2006). Taken together, these experiments revealed a predominantly localization of FUS at excitatory synapses of hippocampal neurons.

Intriguingly, subfractionation experiments using whole brain tissue confirmed that FUS is also present in cellular compartments outside the nucleus including an enrichment in the PSD fraction (Aoki et al., 2012). In our study, FUS was clearly seen in the synaptosomal fraction that contains the complete presynaptic terminal, including mitochondria and synaptic vesicles, the postsynaptic membrane and the PSD. Only a faint signal appeared in the PSD fraction. Both, biochemical fractionations and conventional fluorescence microscopy are, however, methods that are not precise enough to clearly distinguish between the pre- and postsynaptic subcompartments.

### FUS is Localized in the Presynaptic Compartment in Vicinity to Synaptic Vesicles

**FIGURE 6:**
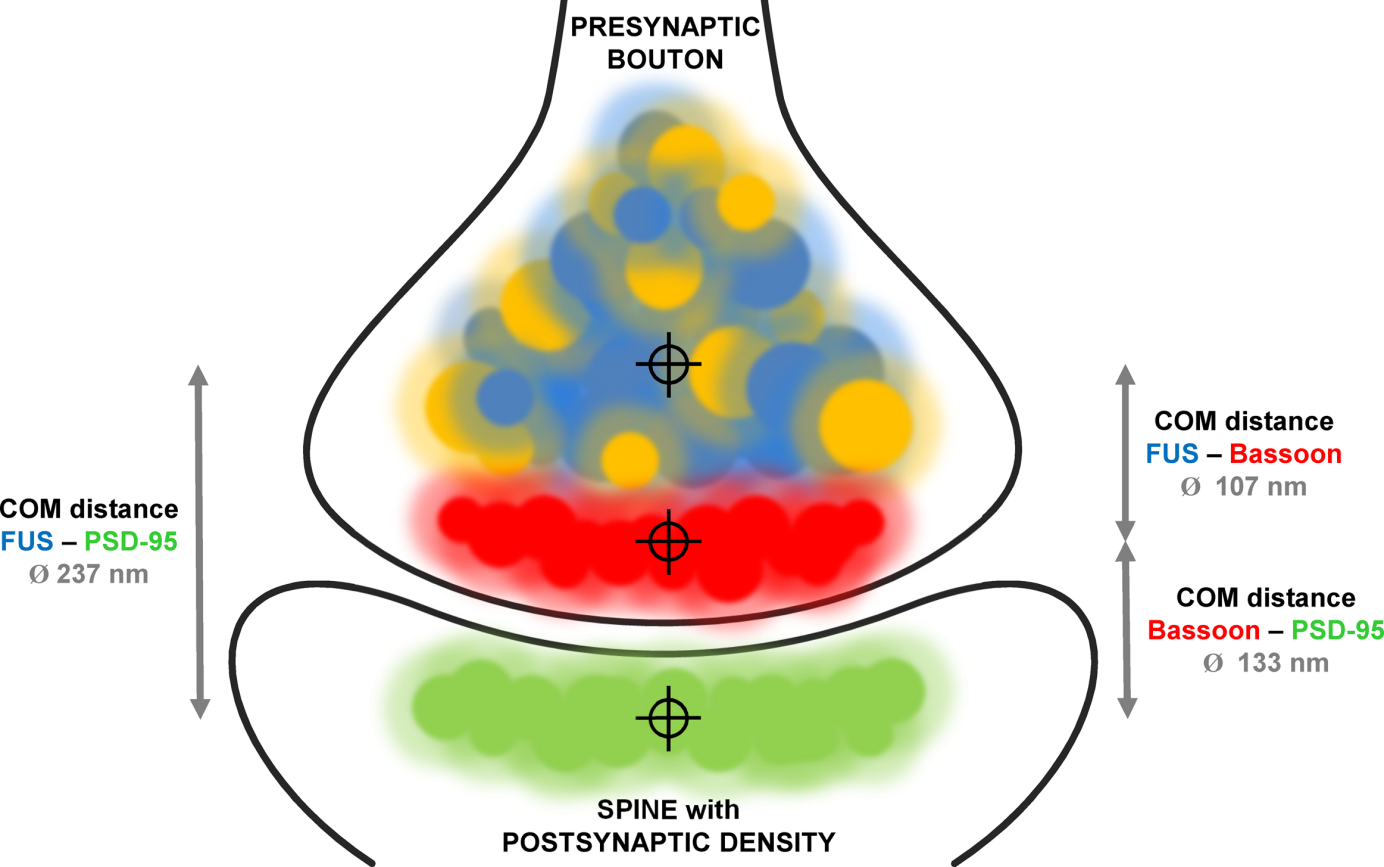
**Model of FUS distribution in relationship to synaptic markers in hippocampal excitatory neurons.** The model displays an excitatory glutamatergic synapse of hippocampal neurons. Bassoon (red) is a typical presynaptic marker while PSD-95 (green) marks postsynaptic densities of synapses using glutamate as neurotransmitter. Co-stainings of FUS (blue) with both markers revealed a presynaptic localization proximal to Bassoon. This was performed by determining the distances (gray arrows) with measured mean values as described in the text between the respective COMs of each protein population (crosshairs). FUS and SYP1 (orange) are highly co-localizing.

We established super-resolved SMLM as an adequate method to determine FUS localization within synapses. By co-staining two pre- and two postsynaptic markers as well as a pre- and postsynaptic marker in parallel we validated our concept of measurement. Our findings are in good agreement with previous studies that investigated the distance of synaptic markers in sections of mouse olfactory bulbs (Dani et al., 2010). We then co-stained (1) FUS with the presynaptic marker Bassoon and (2) FUS with the postsynaptic marker PSD-95. The mean distance between FUS and Bassoon was in the range of the distance between Bassoon and PSD-95 leading to two different possibilities for the localization of FUS: toward the postsynaptic side or more proximal than Bassoon in the direction of the corresponding axon. The distance between FUS and PSD-95 was close to the sum of the distances of FUS/Bassoon and Bassoon/PSD95, revealing a localization on the presynaptic side more distant from the synaptic cleft than Bassoon. A model of the proposed distribution of FUS in cultured hippocampal neurons is depicted in **Figure 6**. The choice of a cutoff that determines which distances should be included into the analysis influences the absolute values of mean distances, but it does not change the conclusion of FUS being presynaptic.

Considering the obtained results of FUS being presynaptic and with a distribution that was broad and rather in the orientation of the synaptic axis – being reminiscent of the typical cone shape, which appears triangular in 2D, of a presynaptic terminal – we hypothesized not only a presynaptic presence of FUS but more precisely a localization of FUS at synaptic vesicles. These abundant structures in the presynapse with a typical size of approximately 40 nm are at the resolution limit of our localization data (Qu et al., 2009). Notably, in our super-resolution images the co-localization of FUS and SYP1 appeared to be highly independent of the presumable synapse orientation since most synapses showed a good overlap of FUS and SYP1. Intriguingly, when having a closer look on the distribution of the two populations at a synapse, they seem to be separated on a few tens of nm-scale. This can be due to several reasons: (1) One explanation would be an antibody interference that might become visible at this length scale. This is supported by the observation that the labeling of FUS and SYP1 seems to behave different in other antibody combinations. (2) Another possibility is that this distribution represents the real situation with little co-localization on a nanoscale level. Further studies are required to distinguish between these models but this is beyond the scope of the current investigations.

Minor discrepancies in our study have to be discussed when comparing the super-resolved SMLM results with the biochemical subfractionation experiments. On the one hand, the microscopy data indicated only a presynaptic localization. We could not observe a weaker second ‘peak’ in the intensity profiles in proximity to PSD-95 signals, however, a postsynaptic targeting of FUS in low amounts cannot be ruled out. On the other hand, the results from the biochemical experiments showed a weak signal of FUS in rat PSD fraction that supposedly only contains postsynaptic densities. Minor ‘contaminations’ of other cellular compartments, especially from the presynapse, might also be considered. Supposing a ‘true’ signal in the PSD fraction and in parallel considering FUS being presynaptic in hippocampal neurons, several reasons could explain such a discrepancy: (1) FUS localization in neurons could be cell-type specific and hence a subfractionation of whole brain lysate could lead to a signal at the postsynaptic side. Contradictory results to the study from Aoki et al. (2012) describing the presence of FUS in higher amounts in the PSD containing fraction compared to the presynaptic compartment from whole mouse brain lysate, would be in line with this hypothesis. To date, it is not possible to rule out a species-dependent FUS localization in the CNS from different rodents. (2) FUS expression and localization could depend on the developmental age of neurons. Our results display a development-dependent expression of the protein. For SMLM, we analyzed cultured hippocampal neurons from embryos 14 days after seeding. This stage resembles more a neuronal maturation stage with ongoing synaptogenesis (Grabrucker et al., 2009) while the biochemical analysis was performed on adult brain material.

### Summary and Outlook

Given its localization in synapses of the mature rat brain, FUS may play an additional role in synapse formation or function. FUS binds mRNAs encoding actin-related proteins, suggesting a role in mRNA transport (Fujii and Takumi, 2005). In this respect, the presynaptic localization of FUS in hippocampal neurons adds another RNA-binding protein localized in the presynaptic compartment. In 2003, SMN and also a member of the hnRNP family (hnRNP R) were localized within axons of motor neurons regulating most likely the translation of beta-actin mRNA (Rossoll et al., 2003). Mutations in SMN are supposed to be causative for spinal muscular atrophy (Jablonka and Sendtner, 2003). In addition, the fragile X mental retardation protein FMRP, that has been intensely characterized with respect to its role in local translation within the postsynaptic compartment, was also found in axons of hippocampal neurons (Antar et al., 2006). Finally, TDP-43, another ALS related RNA-binding protein, could be detected in the axonal compartment of motor neurons (Fallini et al., 2012). Therefore, several RNA-binding proteins are most likely part of a presynaptic machinery that regulates local translation according to specific demands. Since SMN as well as TDP-43 and FUS are known to be mutated in defined diseases of the muscular-skeletal system such as ALS and/or in cortical degenerative disorders leading to FTD, an aberrant function of these proteins at (pre-)synaptic sites has to be taken into consideration with respect to a common pathophysiological mechanism.

In summary, defined mRNA-binding proteins are known to be key player of neuronal mRNA production and transport to fine tune synaptic plasticity (Liu-Yesucevitz et al., 2011). Our study gives insights into the distribution and expression patterns of FUS in various neuronal systems and the primary hippocampal cell culture model. We could not only confirm the nuclear localization of FUS during brain development but showed an additional and precise localization of the protein at the presynaptic terminal of hippocampal excitatory synapses. This comprehensive analysis will foster further functional studies on the role of FUS at synaptic sites in health and disease.

Moreover, we could show an almost complete co-localization of FUS with a synaptic vesicle marker at the scale of the resolution limit of our SMLM data. Further investigations will be needed to describe this putative association on a structural and biochemical level to define binding partners and presynaptic sub-localizations. On a functional level it will be of enormous interest to investigate the putative function of FUS in association with synaptic vesicles. In this respect, it is most interesting that TDP-43 pathology in individuals with sporadic ALS seems to spread along synaptic contacts (Braak et al., 2013). On the same lines, TDP-43 aggregates isolated from brain tissue of ALS and FTD patients induced accumulation of the protein in a prion-like manner in a cell line expressing TDP-43 *in vitro* (Nonaka et al., 2013). Similarly, ALS-associated mutations in FUS were shown to recruit wild-type FUS into pathologic aggregates (Nomura et al., 2014). This mechanism of protein spreading was startlingly also observed in ALS-associated mutations of SOD1 (superoxide dismutase 1) and TDP-43 (Furukawa et al., 2011; Münch et al., 2011; Maniecka and Polymenidou, 2015). Given the described highly prion-like property of FUS, a prion replication mechanism in pathological states should be discussed and could explain the observed spreading behavior (King et al., 2012). In combination with a putative interaction of FUS with synaptic vesicles, which belong to a highly effective signal transduction machinery, it could mean that pathologic FUS might be able to surmount the presynaptic membrane and with an unknown mechanism it might enter subsequent neuronal cells.

## Author Contributions

TB, SP, MS, JM, and MD designed and outlined this study. MD and SP carried out all experiments in cell lines, tissue immunohistochemistry and western blots. Subfractionation experiments and preparation of different brain regions were done by MJS and SP. MS conducted the immunostainings for conventional microscopy as well as for SMLM studies. Optimization of the labeling protocol was done by MS, JR and DD. JR conducted the SMLM measurements and processed the data for image reconstruction. MS analyzed the conventional images statistically; MS and JR jointly analyzed the SMLM images. MS composed most parts of the figures, especially concerning super-resolved and conventional fluorescence imaging of primary neurons. TB, SP, MS, MD, MJS, AL, CP, SL, JR and JM jointly wrote the manuscript.

## Funding

This work was supported by the Helmholtz Gesellschaft (“RNA Dysmetabolism in ALS and FTD” to TB, SP, MS, and SL), the BMBF (“MND-NET” to TB and AL), the Deutsche Forschungsgemeinschaft (DFG SFB 1149-A02 and BO1718/4-1 to TB) and from the European Community’s Health Seventh Framework Programme (FP7/2007-2013) under grant agreement n° 259867 (to AL). Sponsors had no influence on study design or the collection, analysis and interpretation of data.

## Conflict of Interest Statement

The authors declare that the research was conducted in the absence of any commercial or financial relationships that could be construed as a potential conflict of interest.

## Abbreviations

COM: center of mass
DAPI: 4′,6-diamidino-2-phenylindole
dSTORM: direct stochastic optical reconstruction microscopy
FTD: frontotemporal dementia
FUS: Fused in Sarcoma
MAP2: microtubule-associated protein 2
PBS: phosphate buffered saline
PSD: postsynaptic density
PSD-95: postsynaptic density protein 95
SMLM: single molecule localization microscopy
SYP1: Synaptophysin1
TDP-43: Tar DNA-binding protein 43
