# Supplementary material for: Super-Resolution Microscopy Reveals Presynaptic Localization of the ALS/FTD Related Protein FUS in Hippocampal Neurons
Source: Front Cell Neurosci. 2016 Jan 12;9:496. doi: 10.3389/fncel.2015.00496 (PMC4709451; doi:10.3389/fncel.2015.00496)
Supplement: Supplementary file 1 [file Data_Sheet_1.PDF]

Supplementary Material

Super-resolution microscopy reveals presynaptic localization of the ALS / FTD related protein FUS in hippocampal neurons

Michael Schoen[1], Jochen M. Reichel[3], Maria Demestre[1], Stefan Putz[1,2], Dhruva Deshpande[3], Christian Proepper[1], Stefan Liebau[4], Michael J. Schmeisser[1,2], Albert C. Ludolph[2], Jens Michaelis[3*] and Tobias M. Boeckers[1*]

[1]Institute for Anatomy and Cell Biology, Ulm University, Ulm, Germany
[2]Department of Neurology, Ulm University, Ulm, Germany
[3]Institute of Biophysics, Ulm University, Ulm, Germany
[4]Institute of Neuroanatomy, Eberhard Karls University Tübingen, Tübingen, Germany

[*] **Correspondence:** Tobias M. Boeckers, Institute of Anatomy and Cell Biology, Ulm University, Albert-Einstein-Allee 11, Ulm, 89073, Germany, tobias.boeckers@uni-ulm.de
Jens Michaelis, Institute of Biophysics, Ulm University, Albert-Einstein-Allee 11, Ulm, 89073, Germany, jens.michaelis@uni-ulm.de

Supplementary Figures

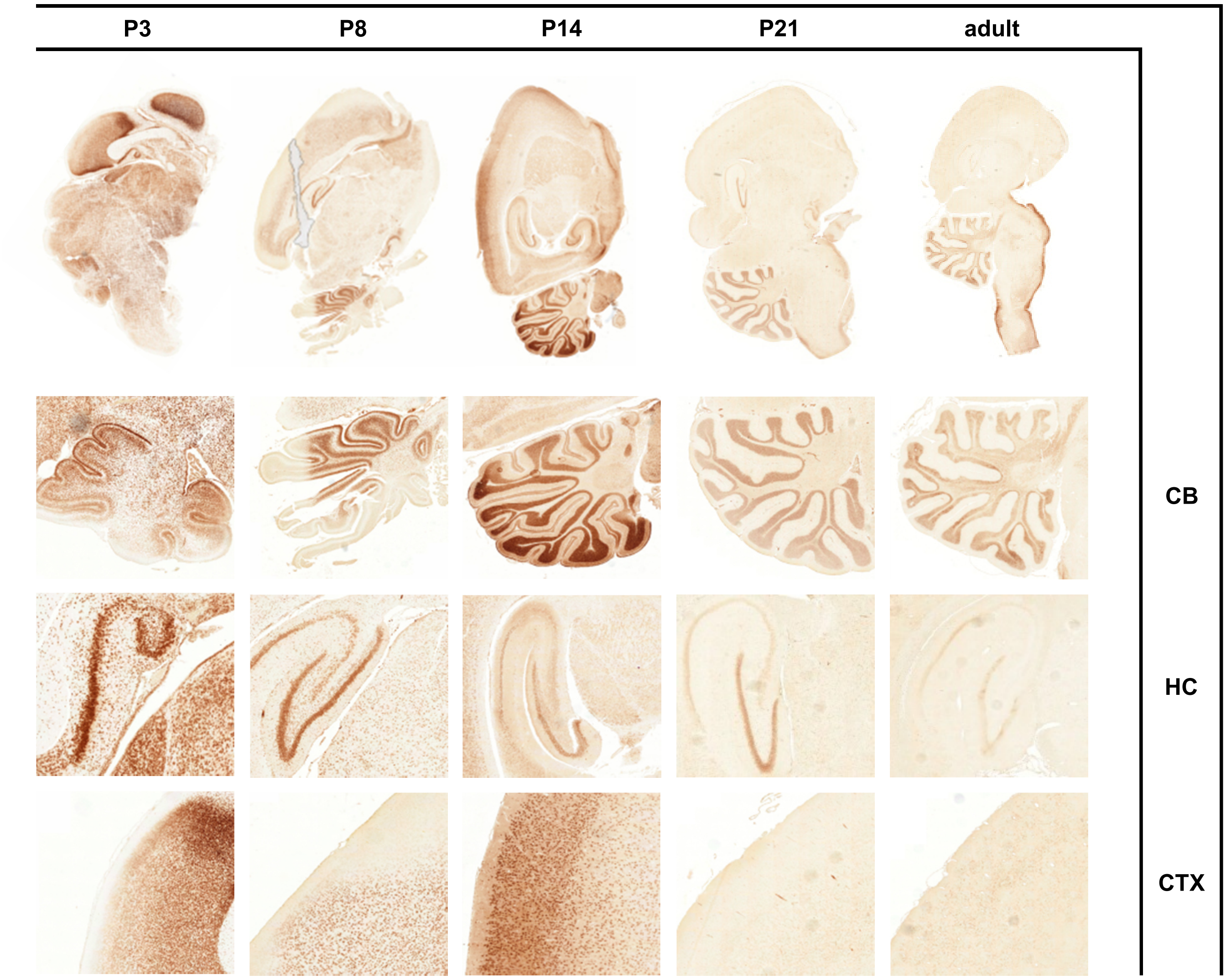

Supplementary Figure S1: Expression of FUS during rat brain development
DAB immunostainings of FUS in different parts of the brain during development (postnatal day P3 to adult) show a clear localization of FUS in all developmental stages and in all areas of the brain. Note that FUS detection was very strong at P14, especially in cortex, hippocampus and cerebellum, and both proteins declined greatly in adult mice, especially in the cortex and hippocampus. CB = cerebellum, HC = hippocampus, CTX = cortex.

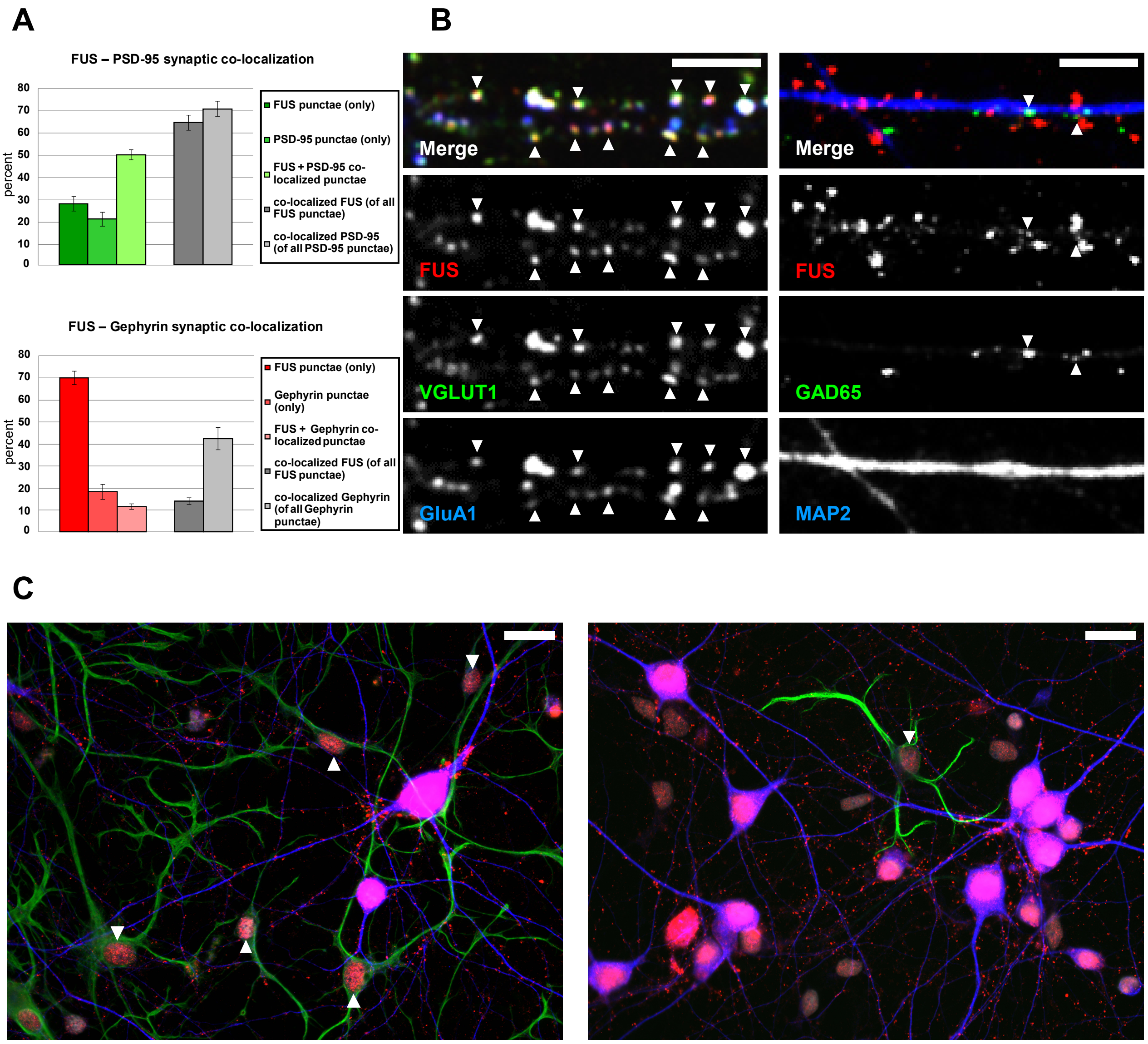

Supplementary Figure S2: Localization of FUS in neuronal and glial cells

(**A**) FUS localization with inhibitory and excitatory synapse markers. Upper graph resulted from evaluation of a co-staining FUS plus PSD-95, lower graph shows FUS plus Gephyrin. Statistical analysis was conducted on min. 10 cells per condition from three independent hippocampal cell preparations fixated 14 days after seeding. Spots and dendrite surface analysis was performed using Imaris software (Bitplane, RRID:nif-0000-00314, SCR_007370). First, dendrites (labeled with MAP2) were transformed into surfaces and subsequently an area of 3 µm around the surface and the area within the surface were defined for spot localization. Secondly, spots were only then considered as co-localizing when having a max. value of 0.7 µm distance between the centers of the respective spots. Error bars represent standard error of the mean. (**B**) Depicted dendrites of hippocampal neurons 15 days after seeding. FUS almost completely co-localizes with the glutamatergic pre- and postsynaptic markers VGLUT1 and GluA1 (left panel) while it overlaps less with the GABAergic presynaptic marker GAD65 (right panel). White arrowheads mark overlaps of synaptic markers with FUS. Scale bars represent 5 µm. (**C**) Besides nuclear and somatodendritic localization in neurons, FUS (red) can be found in nuclei of glial cells (green, left image GFAP, right image S-100 (ß-subunit)). White arrowheads mark glial cell nuclei positive for FUS. Scale bars represent 20 µm.

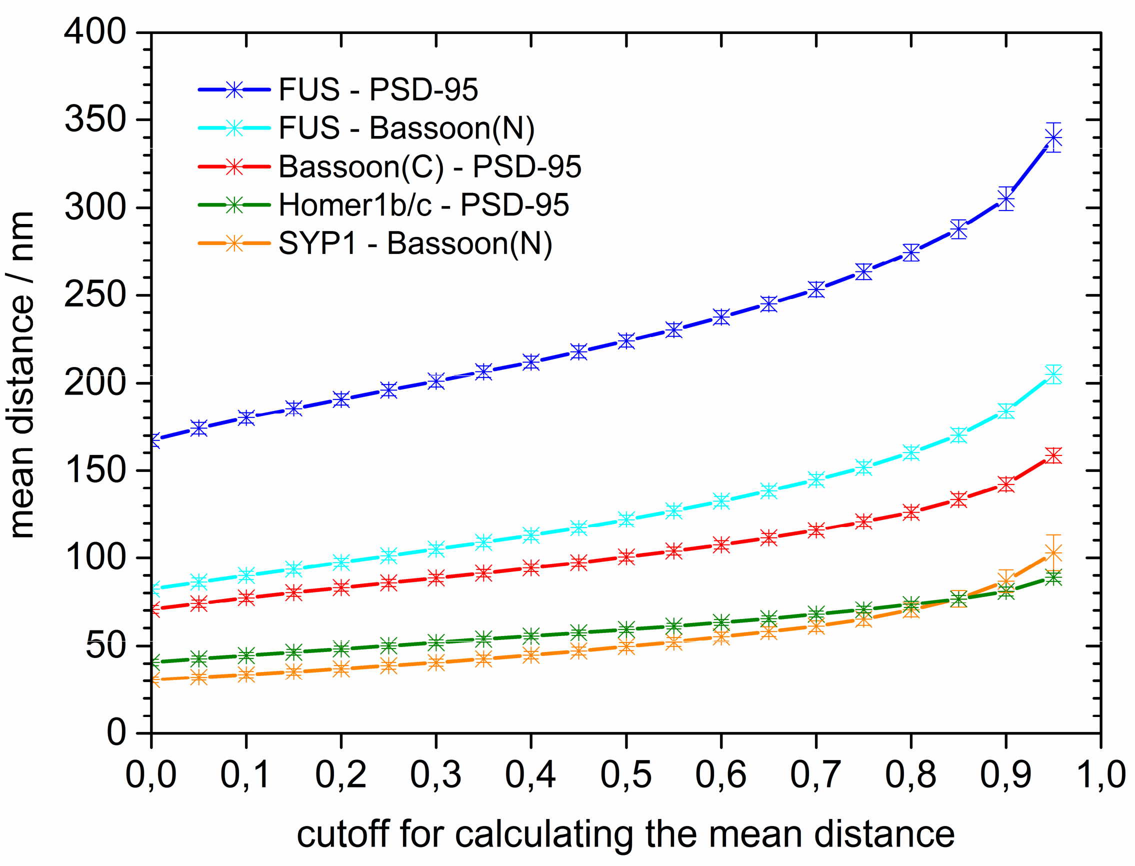

Supplementary Figure S3: Effect of excluding the shortest distances on the mean distance of proteins

Mean distances between respective markers are depicted as a function of inclusion of all or selective synapses. The x-axis reaches from 0.0 (all synapses included) to 0.95 (95 % of all synapses with the shortest COM distance are excluded). Error bars represent standard error of the mean.
